# Effects of Dark Septate Endophytes on the Performance of *Hedysarum scoparium* Under Water Deficit Stress

**DOI:** 10.3389/fpls.2019.00903

**Published:** 2019-07-11

**Authors:** Xia Li, Xue-Li He, Yong Zhou, Yi-Ting Hou, Yi-Ling Zuo

**Affiliations:** ^1^College of Life Science, Hebei University, Baoding, China; ^2^College of Landscape Architecture and Tourism, Hebei Agricultural University, Baoding, China

**Keywords:** *Hedysarum scoparium*, dark septate endophytes, water deficit stress, non-host endophytes, inoculation

## Abstract

*Hedysarum scoparium*, a species characterized by rapid growth and high drought resistance, has been used widely for vegetative restoration of arid regions in Northwest China that are prone to desertification. Desert soil is typically deficient in available water and the alleviation of drought stress to host plants by endophytes could be an efficient strategy to increase the success of desert restoration. With the objective to seek more beneficial symbionts that can be used in the revegetation strategies, we addressed the question whether *H. scoparium* can benefit from inoculation by dark septate endophytes (DSEs) isolated from other desert plants. We investigated the influences of four non-host DSE strains (*Phialophora* sp., *Knufia* sp., *Leptosphaeria* sp., and *Embellisia chlamydospora*) isolated from other desert plants on the performance of *H. scoparium* under different soil water conditions. Differences in plant performance, such as plant growth, antioxidant enzyme activities, carbon, nitrogen, and phosphorous concentration under all the treatments, were examined. Four DSE strains could colonize the roots of *H. scoparium* successfully, and they established a positive symbiosis with the host plants depending on DSE species and water availability. The greatest benefits of DSE inoculation occurred in water stress treatment. Specifically, *Phialophora* sp. and *Leptosphaeria* sp. improved the root biomass, total biomass, nutrient concentration, and antioxidant enzyme activities of host plants under water deficit conditions. These data contribute to the understanding of the ecological function of DSE fungi in drylands.

## Introduction

Approximately 27% of the land area in China is exposed to desertification, causing critical ecological and environmental problems, especially in Northwest China (Tang et al., 2016). The Chinese government has implemented a variety of solutions, including afforestation projects, to reduce the effect of desertification (Su et al., 2007; Fan et al., 2016). Over the past few decades, restoration of deserts using xerophyte shrubs is viewed as a common and effective method in many arid regions (Zhu and Chen, 1994; Wang et al., 2012; Deng et al., 2015). These plants have evolved various mechanisms, including altered morphological and physiological properties, to cope with drought stress (Deng et al., 2015; Li et al., 2017). Plants in natural habitats often harbor ubiquitous fungal endophytes, some of which are drought-tolerant and can stimulate plant growth in arid ecosystems (Ren et al., 2011; González-Teuber et al., 2018; Xie et al., 2018). Mitigating drought stress in host plants by endophytes may be an efficient strategy to improve the restoration rate of desert soils, which are typically deficient in available water (Gong et al., 2015; Shi et al., 2015; González-Teuber et al., 2018).

Most desert plants associate with a wide diversity of root endophytic fungi, including dark septate endophytes (DSEs) (Barrow, 2003; Porras-Alfaro et al., 2008; González-Teuber et al., 2017). These endophytes are characterized by melanized septate hyphae and microsclerotia and are found in the roots of more than 600 plant species (Jumpponen and Trappe, 1998). DSE are abundant root colonists especially in plants growing under extreme conditions such as arid environments (Jumpponen and Trappe, 1998; Barrow and Osuna, 2002). Many researchers have investigated and isolated a variety of DSE from grasses, shrubs, and trees in arid areas (Lugo et al., 2009, 2015; Knapp et al., 2012, 2015; Li et al., 2015, 2018; Xie et al., 2017). For instance, Lugo et al. (2015) investigated DSE in 42 plants from an arid region in Argentina and showed that DSE were frequently present in the roots. In Northwest China, DSE were also observed and identified in the roots of multiple desert shrubs, such as *Ammopiptanthus mongolicus*, *Hedysarum scoparium*, and *Gymnocarpos przewalskii* (Li et al., 2015, 2018; Xie et al., 2017). For example, in our previous survey conducted in seven arid and semi-arid locations in Northwest China, we showed that *H. scoparium* was highly colonized by DSE and we isolated nine DSE species from their roots (Xie et al., 2017). However, our understanding of DSE functions in relation to plants is still limited (Barrow, 2003).

Although not all DSE–plant relationships are beneficial, there is a strong evidence to suggest that DSE may positively influence plant resistance to drought by increasing plant growth, water and nutrient absorption, and/or facilitating plant resistance to oxidation stress (Perez-Naranjo, 2009; Santos et al., 2017). In field experiments, Barrow (2003) investigated the DSE of native grasses in arid southwestern United States rangelands. The author proposed that DSE may form a continuous integrated network that enhances nutrient and water transport in roots. DSE are readily isolated and cultured *in vitro*, which has facilitated the studies on the effects of DSE on host plants under water stress in controlled culture conditions. In pot experiments, desert plants *Agropyron cristatum* and *Psathyrostachys juncea* inoculated with DSE developed a higher shoot biomass and carbon content compared with non-inoculated plants under drought conditions (Perez-Naranjo, 2009). Similarly, Santos et al. (2017) conducted an inoculation experiment to study the influence of DSE isolates on rice under water deficit induced with polyethylene glycol 6000. Their results showed that DSE increase the growth and decrease the oxidative stress in rice plants. However, this positive effect occurred only in specific water stress conditions.

*Hedysarum scoparium*, a species characterized with rapid growth and high drought resistance, is a pioneer desert shrub that has been widely used for prevention of desertification and vegetative restoration in arid and semiarid regions of China (Hu et al., 2009). In a pot experiment conducted in our study, DSE isolated from healthy roots of *H. scoparium*, colonized the host roots and increased the shoot and root biomass of *H. scoparium* plants (Supplementary Figure 1). With the objective to seek more beneficial symbionts that can be used in revegetation strategies, we investigated whether *H. scoparium* can benefit from DSE isolated from other desert plants. Four DSE isolated from the roots of *G. przewalskii*, a species with similar growth habit and ecological distribution to that of *H. scoparium*, were selected for the inoculation experiment. These fungi colonized the roots of other desert plants with no apparent disease symptoms and enhanced plant growth under drought conditions (Li et al., 2018). In this study, we aimed to evaluate the effects of non-host DSE inoculation on *H. scoparium* plants growing in drought sandy soil under greenhouse conditions. We focused on plant growth, nutrient content, and activity of antioxidant enzymes to address the following questions: (1) Do DSE from other plants colonize the roots of *H. scoparium* under well-watered and water deficit conditions? (2) If yes, can these non-host DSE improve the growth and physiological performance of *H. scoparium* plants? (3) Does water availability affect the symbiosis-dependent benefits? (4) How non-host DSE help *H. scoparium* plants to overcome water deficit stress?

## Materials and Methods

### Fungal Isolates and Plant Materials

Four DSE fungi isolated from the roots of *G. przewalskii*, which grows naturally in extreme arid deserts of Northwest China, were used in this experiment. Their species identification was confirmed previously through internal transcribed spacer (ITS) phylogeny (Li et al., 2018). All the fungal isolates belonged to different species and they included: *Phialophora* sp., *Knufia* sp., *Leptosphaeria* sp., and *Embellisia chlamydospora*. These fungi are deposited in the culture collection of the Laboratory of Plant Ecology, Hebei University, China, and their ITS sequences are available from GenBank under accession numbers MF036001 for *Phialophora* sp., MF036003 for *Knufia* sp., MF036004 for *Leptosphaeria* sp., and MF036005 for *E. chlamydospora*.

*Hedysarum scoparium* was chosen as a host plant in this study mostly for its important role in vegetation restoration and known high DSE colonization frequency (Xie et al., 2017). The seeds of *H*. *scoparium* were collected from natural populations in Gansu Province, Northwest China, and stored at 4°C.

### Experimental Design

The experiment was conducted in a growth chamber in a completely randomized factorial design (5 inoculation treatments × 2 water treatments) with five replicates. The inoculation treatments included inoculation with *Phialophora* sp., *Knufia* sp., *Leptosphaeria* sp., and *E. chlamydospora* and a non-inoculated control. The water treatments were well-watered and water deficit stress. A total of 50 pots were prepared.

The seeds of *H. scoparium* were surface-sterilized by dipping in 70% ethanol for 3 min and then in 2.5% sodium hypochlorite for 10 min with agitation. The sterilized seeds were gently washed by sterile water several times and then aseptically planted onto water agar medium (containing 10 g/L agar) in Petri dishes for germination at 27°C. Following pregermination, each seedling was transferred into sterile pot (8 cm diameter, 24 cm height) containing 500 g sand collected from the natural habitats of *H*. *scoparium* and autoclaved for 120 min at 121°C. The basic physicochemical characteristics of the sand were as follows: organic matter 23.17 mg/g, available nitrogen 21.63 mg/kg, and available phosphorus 1.53 mg/kg. One month later, half of the seedlings were maintained under well-watered conditions throughout the entire experiment (70% field water capacity), and the other half were exposed to water deficit stress (30% field water capacity). Water loss was daily supplemented with sterile distilled water to keep the desired field capacity by regular weighing. The water content for water deficit treatment was chosen according to the median value in the natural habitat of *H*. *scoparium* in Northwest China (Xie, 2017).

Fungal inocula were prepared by aseptically growing DSE isolates in Petri dishes with potato dextrose agar culture medium. For DSE inoculation, two 5 mm plugs excised from an edge of an actively growing colony on culture medium were inoculated at a 1 cm range close to the roots of *H*. *scoparium* seedlings. The non-inoculated controls were inoculated with plugs excised from the sterile medium without fungus. All the inoculation processes were carried out on a clean bench, and all the pots were kept in a growth chamber with a 14 h/10 h photoperiod, temperature of 27°C/22°C (day/night), and 60% mean air relative humidity. The duration of the stress experiment was 4 months.

### Harvest of *Hedysarum scoparium* Seedlings

At the end of the experiment, the shoots and roots from each plant were separately harvested, and the roots were gently washed with tap water to remove the sand. Subsamples of fresh roots and shoots were set aside for assessing DSE colonization status and antioxidant enzyme activity, respectively, as described below. The remaining part of shoots and roots were weighed before drying in an oven at 70°C for 48 h and the water content was measured. The biomass production of plants was the sum of the dry weights of these two parts. After that the dried shoot and root materials were ground into a powder to measure the concentrations of carbon (C), nitrogen (N), and phosphorus (P).

### Microscopic Observation of Root Colonization

Root colonization by DSE isolates was evaluated using the method described by Phillips and Hayman (1970). The sampled roots were cleared with 10% KOH in a water bath at 100°C for 60 min and then stained with 0.5% (w/v) acid fuchsin at 90°C for 20 min. Overall, 20 randomly selected 0.5 cm long root segments per sample were placed on slides and observed under an optical microscope.

### Determination of Antioxidant Enzyme Activity

To determine the activity of different antioxidant enzymes, fresh leaf samples from each plant were homogenized in 5 mL of 50 mM potassium phosphate buffer (pH 7.8), which contained chilled 0.2 mM EDTA and 2% (w/v) polyvinylpyrrolidone kept in ice bath. Prechilled mortar and pestle were used for grinding. The homogenate was centrifuged at 15,294 × *g* and 4°C for 30 min. The supernatant was decanted and used for analysis of enzymes.

The superoxide dismutase (SOD) activity was determined using the photochemical method described by Elavarthi and Martin (2010) by recording the decrease in the absorbance of nitro blue tetrazolium complex due to its reduction by the enzyme. One unit of SOD was equivalent to the quantity of enzyme needed to inhibit the reduction rate of NBT by 50% at a wavelength of 560 nm. The catalase (CAT) activity was determined by measuring the consumption of H_2_O_2_ at 240 nm wavelength for 1 min. The reaction mixture consisted of 25 mM potassium phosphate buffer (pH 7.0), 10 mM H_2_O_2_, and enzyme extract (Cakmak and Marschner, 1992).

### Carbon, Nitrogen, and Phosphorus Concentrations

The C and N concentrations in the shoots and roots were directly determined using the dry combustion method with an elemental analyzer (Vario EL/micro cube; Elementar, Hanau, Germany). For determination of P concentrations, dried ground shoot and root samples were digested in HNO_3_, followed by a microwave-accelerated reaction in a Microwave-Accelerated Digestion System (MARS; CEM, Corp., Matthews, NC, United States). The P concentrations were measured by the molybdenum-antimony colorimetric method (Bao, 2000).

### Statistical Analysis

All statistical analyses were performed with SPSS software (Version 21; SPSS, Chicago, IL, United States). A two-way analysis of variance was used to analyze the effects of DSE inoculation, water treatment, and their interaction on plant biomass, leaf antioxidant enzyme activity, and element concentrations in the roots and shoots. All data were tested for normality and homogeneity of variance before statistical analyses. The differences between the means among different treatments were compared using Duncan’s multiple-range tests at *P* < 0.05.

## Results

### DSE Root Colonization

After harvesting, no DSE structures were detected in the roots of control plants regardless of water treatment, while the presence of DSE hyphae and microsclerotia was confirmed in stained root segments of inoculated plants (Supplementary Figure 2).

### Plant Biomass Production

**FIGURE 1:**
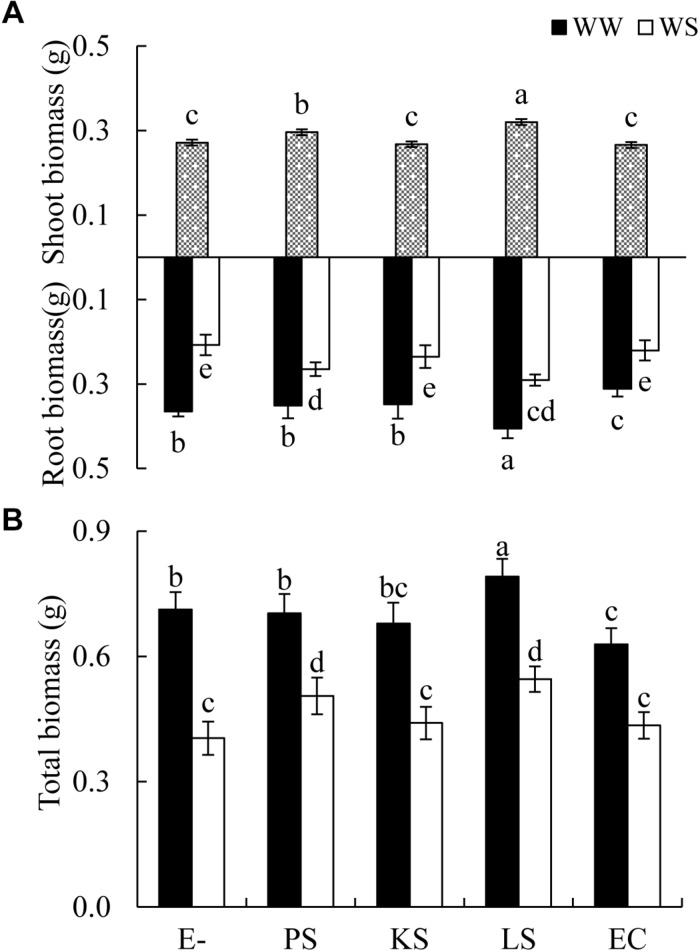
Effects of dark septate endophyte (DSE) inoculation and water treatment on the biomass production of *Hedysarum scoparium*. **(A)** Shoot and root biomass of *Hedysarum scoparium*. **(B)** Total biomass of *Hedysarum scoparium*. The error bars represent the standard error (SE). Different letters above the error bars indicate significant difference at *P* < 0.05 by Duncan’s multiple-range tests. Since DSE × water treatment interactions were not significant for shoot biomass, means for the main factor (DSE) were presented. E– indicates non-inoculated plants. PS, KS, LS, and EC indicate plants inoculated with *Phialophora* sp., *Knufia* sp., *Leptosphaeria* sp., and *Embellisia chlamydospora*, respectively. WW and WS indicate well-watered and water stress treatment.

The shoot biomass of *H*. *scoparium* was affected significantly by the DSE inoculation regardless of the water regime (Table 1). Specifically, the inoculation of *Phialophora* sp. and *Leptosphaeria* sp. resulted in significant increases in shoot biomass (by 9.0 and 17.9%, respectively) compared to control plants (Figure 1).

**TABLE 1:** Analysis of variance for the effects of DSE inoculation and water stress treatment on biomass production of *Hedysarum scoparium*.

	**Shoot biomass (g)**		**Root biomass (g)**		**Total biomass (g)**	
	***F***	***P***	***F***	***P***	***F***	***P***
DSE	11.6	**<0.001**	18.0	**<0.001**	17.2	**<0.001**
Water stress	406.7	**<0.001**	300.6	**<0.001**	420.7	**<0.001**
DSE × Water stress	1.8	0.156	3.8	**0.011**	3.2	**0.023**

*Significant *P*-values are in bold.*

The interactions of DSE inoculation and water treatment were significant for both the root and total biomass of *H*. *scoparium* seedlings (Table 1). Under well-watered conditions, inoculation with *Leptosphaeria* sp. led to a significantly greater root and total biomass (40.0 and 35.1%, respectively) compared with those of the control plants, whereas the inoculation with *E. chlamydospora* resulted in a 14.8 and 11.7% decrease in both parameters, respectively (Figure 1). Under water stress conditions, *Phialophora* sp. and *Leptosphaeria* sp. inoculations caused a significantly greater root (27.5 and 40.0%) and total biomass (25.1 and 35.1%) compared with those observed in control plants, respectively. There was no significant difference in the root and total biomass production among plants inoculated with *Knufia* sp. and *E. chlamydospora* and the control (Figure 1).

### Antioxidant Enzyme Activities in Leaves of *Hedysarum scoparium*

**FIGURE 2:**
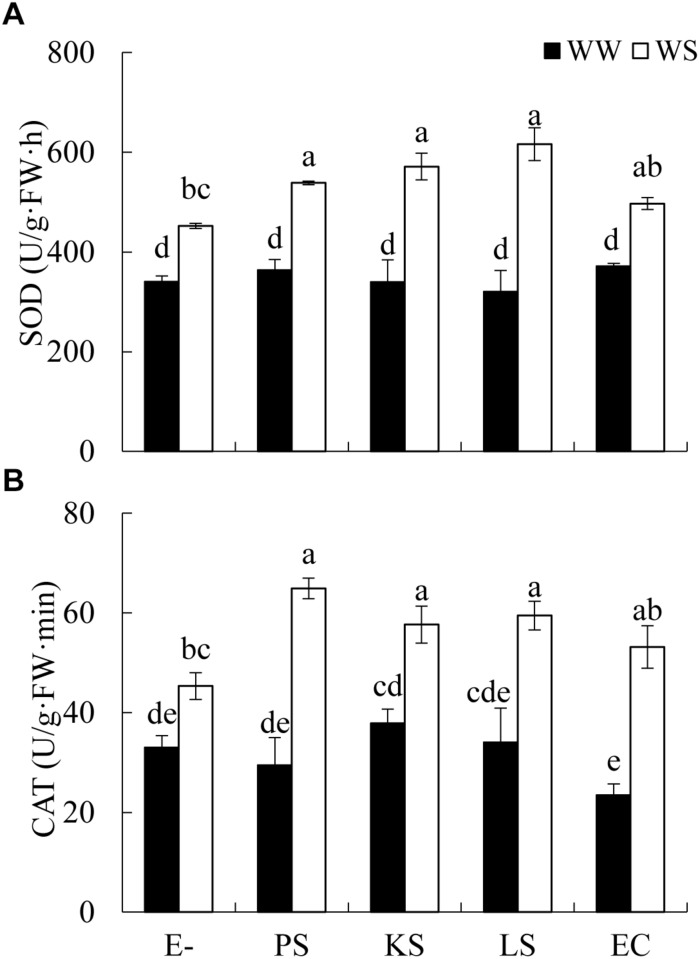
Effects of DSE inoculation and water treatment on the antioxidant enzyme activities in leaves of *Hedysarum scoparium*. **(A)** Superoxide dismutase (SOD) in leaves of *Hedysarum scoparium*. **(B)** Catalase (CAT) in leaves of *Hedysarum scoparium*. The error bars represent the SE. Different letters above the error bars indicate significant difference at *P* < 0.05 by Duncan’s multiple-range tests. E– indicates non-inoculated plants. PS, KS, LS, and EC indicate plants inoculated with *Phialophora* sp., *Knufia* sp., *Leptosphaeria* sp., and *Embellisia chlamydospora*, respectively. WW and WS indicate well-watered and water stress treatment.

The interaction between DSE inoculation and water treatment showed significant effects on the antioxidant enzyme activities in leaves of *H*. *scoparium* (Table 2). In general, all the tested DSE caused remarkable increases in the activities of SOD and CAT under water stress conditions, which was closely related to the drought tolerance of *H*. *scoparium* (Figure 2). Plants inoculated with *Phialophora* sp., *Knufia* sp. and *Leptosphaeria* sp. showed significantly higher values of SOD (19.1, 26.4, and 36.4%) and CAT (43.2, 27.2, and 31.1%, respectively) activities compared with control plants under water stress conditions. However, under well-watered conditions, there were no significant differences in either SOD or CAT activity between DSE inoculated plants and control plants. *E. chlamydospora* inoculation had no significant effects on host plants compared with control plants under both water regimes (Figure 2).

**TABLE 2:** Analysis of variance for the effects of dark septate endophyte (DSE) inoculation and water stress treatment on the antioxidant enzyme activities and element concentration of *Hedysarum scoparium*.

	**SOD (U/g⋅FW⋅h)**		**CAT (U/g⋅FW⋅min)**		**Shoot C (mg/g)**		**Root C (mg/g)**		**Shoot N (mg/g)**		**Root N (mg/g)**		**Shoot P (mg/g)**		**Root P (mg/g)**	
	***F***	***P***	***F***	***P***	***F***	***P***	***F***	***P***	***F***	***P***	***F***	***P***	***F***	***P***	***F***	***P***
DSE	2.4	0.064	2.9	**0.033**	2.3	0.071	0.4	0.775	6.4	**<0.001**	1.7	0.167	2.6	0.052	2.4	0.070
Water stress	137.6	**<0.001**	101.2	**<0.001**	127.8	**<0.001**	14.4	**<0.001**	152.6	**<0.001**	29.7	**<0.001**	10.2	**0.003**	11.9	**0.001**
DSE × Water stress	4.6	**0.004**	2.7	**0.046**	3.1	**0.026**	0.4	0.798	3.4	**0.018**	2.2	0.091	0.3	0.856	0.3	0.905

*C, carbon; N, nitrogen; P, phosphorus; SOD, superoxide dismutase; CAT, catalase; FW, fresh weight. Significant *P*-values are in bold.*

### Element Concentration in Plant Tissues

**FIGURE 3:**
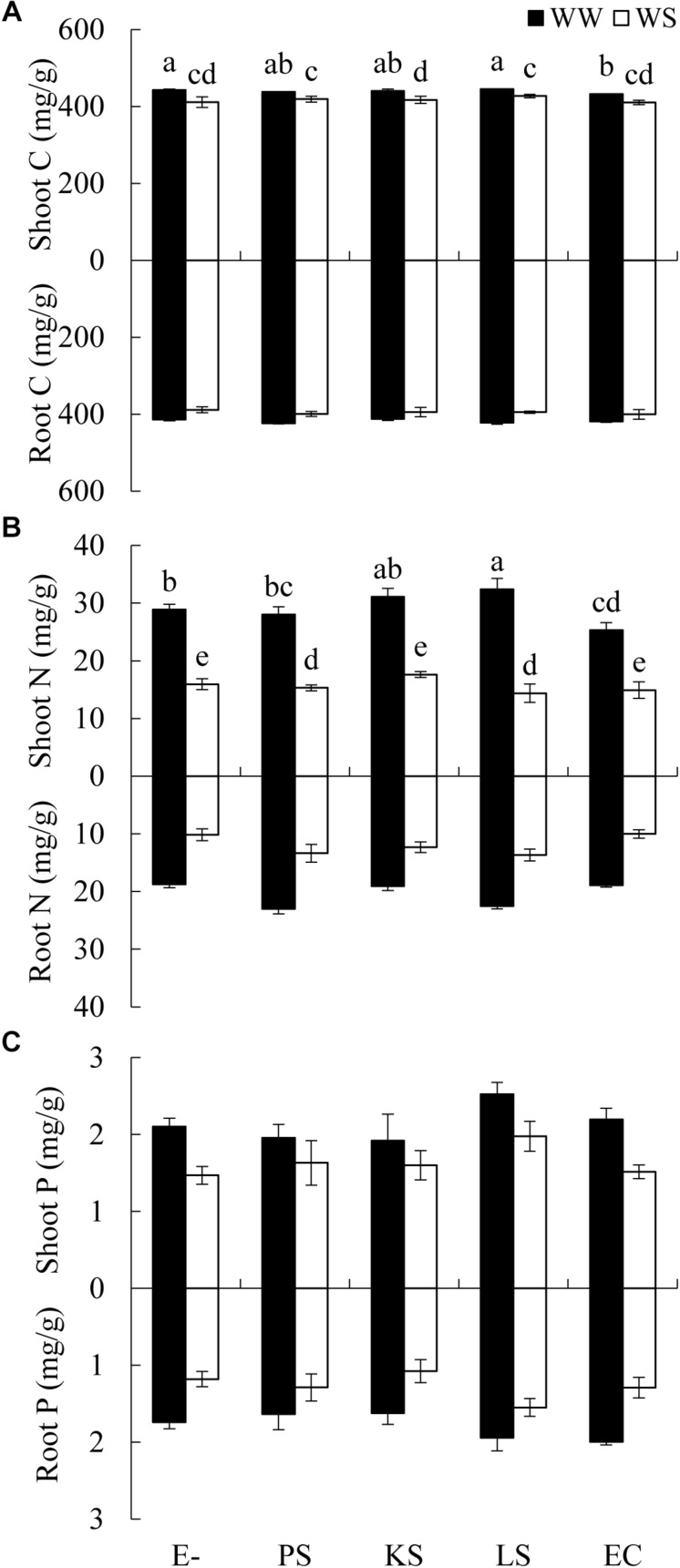
Effects of DSE inoculation and water treatment on the elemental concentration of *Hedysarum scoparium*. **(A)** Shoot and root carbon concentration (C) of *Hedysarum scoparium*. **(B)** Shoot and root nitrogen concentration (N) of *Hedysarum scoparium*. **(C)** Shoot and root phosphorus concentration (P) of *Hedysarum scoparium*. The error bars represent the SE. Different letters above the error bars indicate significant difference at *P* < 0.05 by Duncan’s multiple-range tests. The estimated means were presented when interactions were not significant. E– indicates non-inoculated plants. PS, KS, LS, and EC indicate plants inoculated with *Phialophora* sp., *Knufia* sp., *Leptosphaeria* sp., and *Embellisia chlamydospora*, respectively. WW and WS indicate well-watered and water stress treatment.

There was a significant interaction between the DSE inoculation and water treatment on the shoot C concentration of *H*. *scoparium* (Table 2). The shoot C concentration was significantly lower (2.5%) in *E. chlamydospora*-inoculated *H*. *scoparium* plants than in the control plants under well-watered conditions, but there was no significant effect of DSE inoculation on the shoot C content in host plants in all the other treatments (Figure 3A). The N concentration in the shoots of *H*. *scoparium* was affected significantly by the DSE inoculation, water treatment, and their interaction (Table 2). Under water stress conditions, inoculation with *Phialophora* sp. and *Leptosphaeria* sp. resulted in a significant increase in the N concentration in the shoots of *H*. *scoparium* by 22.9 and 20.2%, respectively, when compared with control plants. Under well-watered conditions, *Leptosphaeria* sp. induced a significant increase in the shoot N concentration (12.3%), while *E. chlamydospora* showed an opposite effect, with about 87.7% of control plants (Figure 3B). DSE inoculation did not affect the root C, N, and P concentrations and shoot P concentration of *H*. *scoparium* seedlings under all the treatments (Figure 3 and Table 2).

## Discussion

As important root endophytes, DSE have been reported to have positive ecological roles in plant growth and nutrient uptake (Wu et al., 2010; Newsham, 2011; Surono and Narisawa, 2017; Vergara et al., 2018). They also provide increased plant resistance to a wide range of environmental stressors (Andrade-Linares et al., 2011; Likar and Regvar, 2013; Su et al., 2013; Berthelot et al., 2017; Santos et al., 2017; Zhang et al., 2017; Jin et al., 2018). However, little is known about the relationship of non-host DSE and host plants, especially when water availability is considered (Zhang et al., 2017). In the studies using crops and plants growing in heavy metal-contaminated soils, DSE fungi, which have been considered non-host colonizers, showed the potential to improve plant growth (Li et al., 2011; Khastini et al., 2012; Berthelot et al., 2016, 2017; Wang et al., 2016). For example, *Gaeumannomyces cylindrosporus* isolated from plants naturally growing in an ancient Pb-Zn slag heap were reported to enhance the growth of maize under Cd stress (Ban et al., 2017). Similarly, the four DSE in this study isolated from *G. przewalskii* were able to colonize the roots of *H. scoparium* plants under both well-watered and water deficit conditions. Similarly, Zhang et al. (2017) reported that *Exophiala pisciphila* isolated from maize could colonize the roots of *Sorghum bicolor*. Our results indicated that DSE isolated from desert plants may be used in other plants. Moreover, they exhibited positive effects on shoot C and N content and biomass production of *H. scoparium* plants; however, these effects were strain-dependent. This observation corroborates previous reports that state that DSE fungal species may be one of the factors that influence the symbiotic relationship (Wilcox and Wang, 1987; Mandyam and Jumpponen, 2005; Newsham, 2011). In addition, both DSE strains isolated from *H. scoparium* promoted the shoot and root growth of *H. scoparium* plants under well-watered condition in our previous study (Supplementary Figure 1). This indicates that the outcome of DSE – *H. scoparium* interactions may also depend on the host origin of the DSE involved. Therefore, choosing DSE strains that are most beneficial to the plants during the restoration of desert vegetation is vital.

The present study further demonstrated that the interaction between DSE and *H. scoparium* depended on water availability. Under water deficit conditions, no adverse effects were found in all the plants inoculated with DSE. The biomass production and shoot C and N content of plants inoculated with *Phialophora* sp. and *Leptosphaeria* sp. was increased compared with that of the control, whereas the inoculation of *H. scoparium* plants with *E. chlamydospora* negatively affected plant growth under well-watered conditions, but caused no significant decline in plant growth when exposed to water stress. This indicates that the interaction between DSE and *H. scoparium* becomes positive under water stress. Our results agree with previous studies in other crops, which have shown that the benefits of plant–endophyte associations seem to be stronger under soil water stress conditions (Zhang et al., 2017). For *H. scoparium* plants, benefits of symbiosis under water stress may be advantageous to the plant growth in their natural drought habitats.

The ability of DSE fungi to promote plant growth under water stress conditions may be related to the increased C and N absorption, as well as to the enhanced activities of antioxidant enzymes. Vergara et al. (2018) have reported that increased N absorption by tomato plants was in response to inoculation with DSE isolates—inoculated plants exhibited higher dry weight than non-inoculated plants when supplied with organic N. Similarly, in our study, *Phialophora* sp. and *Leptosphaeria* sp. may have facilitated the absorption of C and N in the shoots of *H. scoparium*. This might be due to the ability of DSE fungi to mineralize organic compounds containing C, N, and P, thereby making them available to plants (Della Monica et al., 2015; Surono and Narisawa, 2017). For example, *Phialocephala fortinii*, a plant growth promoter in many studies, was reported to have the ability to degrade polymeric forms of C, N, and P such as cellulose, starch and protein (Caldwell et al., 2000; Surono and Narisawa, 2017). The increased SOD and CAT activity is another possible mechanism of increased plant growth. Water stress usually exerts negative effects on organisms and causes cellular oxidative damage (Bartels and Sunkar, 2005). SOD and CAT are the primary enzymes involved in the antioxidant system of plants (Khan et al., 2018; Saleem et al., 2018). In the present study, plants inoculated with *Phialophora* sp., *Knufia* sp., and *Leptosphaeria* sp. contained significantly higher concentrations of SOD and CAT compared with control plants under water deficit conditions. These findings can be related with the work by Santos et al. (2017), who found that DSE increased the tolerance of rice plants to water stress through altered antioxidant enzyme activity. However, the mechanisms leading to an increase in the growth of plants inoculated with DSE fungi warrants further research.

## Conclusion

We found that non-host DSE could colonize the roots of *H. scoparium* and benefit the plant growth, through combined mechanisms of increased nutrient absorption and enhanced antioxidant systems, under water deficit conditions. Our results complement previous insight that endophytes can promote drought resistance in plants and highlight the importance of using DSE in desert plants in water-stressed conditions (Kivlin et al., 2013; Shi et al., 2015; Zhang et al., 2017; González-Teuber et al., 2018; Xie et al., 2018). As *H. scoparium* plays important roles in vegetative restoration, the DSE–*H. scoparium* association has the potential for further testing in the field to determine its ability to suppress desertification in arid regions of Northwest China.

## Author Contributions

XL and X-LH conceived and designed the experiments and wrote the manuscript. XL, YZ, Y-TH, and Y-LZ performed the experiments. XL and YZ analyzed the data.

## Conflict of Interest Statement

The authors declare that the research was conducted in the absence of any commercial or financial relationships that could be construed as a potential conflict of interest.

## References

[B1] Andrade-Linares D. R. Grosch R. Restrepo S. Krumbein A. Franken P. (2011). Effects of dark septate endophytes on tomato plant performance. *Mycorrhiza* 21 413–422. 10.1007/s00572-010-0351-1 21184117

[B2] Ban Y. Xu Z. Yang Y. Zhang H. Chen H. Tang M. (2017). Effect of dark septate endophytic fungus *Gaeumannomyces cylindrosporus* on plant growth, photosynthesis and Pb tolerance of maize (*Zea mays* L.). *Pedosphere* 27 283–292. 10.1016/s1002-0160(17)60316-3

[B3] Bao S. D. (2000). *Agrochemical Analysis of Soil.* Beijing: Chinese Agricultural Press.

[B4] Barrow J. R. (2003). Atypical morphology of dark septate fungal root endophytes of *Bouteloua* in arid southwestern USA rangelands. *Mycorrhiza* 13 239–247. 10.1007/s00572-003-0222-0 14593517

[B5] Barrow J. R. Osuna P. (2002). Phosphorus solubilization and uptake by dark septate fungi in fourwing saltbush, *Atriplex canescens* (Pursh) Nutt. *J. Arid. Environ.* 51 449–459. 10.1006/jare.2001.0925

[B6] Bartels D. Sunkar R. (2005). Drought and salt tolerance in plants. *Crit. Rev. Plant Sci.* 24 23–58. 10.1080/07352680590910410

[B7] Berthelot C. Blaudez D. Leyval C. (2017). Differential growth promotion of poplar and birch inoculated with three dark septate endophytes in two trace element-contaminated soils. *Int. J. Phytoremediat.* 19 1118–1125. 10.1080/15226514.2017.1328392 28521510

[B8] Berthelot C. Leyval C. Foulon J. Chalot M. Blaudez D. (2016). Plant growth promotion, metabolite production and metal tolerance of dark septate endophytes isolated from metal-polluted poplar phytomanagement sites. *FEMS Microbiol. Ecol.* 92:fiw144. 10.1093/femsec/fiw144 27364359

[B9] Cakmak I. Marschner H. (1992). Magnesium deficiency and high light Intensity enhance activities of superoxide dismutase, ascorbate peroxidase, and glutathione reductase in bean leaves. *Plant Physiol.* 98 1222–1227. 10.1104/pp.98.4.1222 16668779 PMC1080336

[B10] Caldwell B. A. Jumpponen A. Trappe J. M. (2000). Utilization of major detrital substrates by dark-septate, root endophytes. *Mycologia* 92 230–232. 10.2307/3761555

[B11] Della Monica I. F. Saparrat M. C. N. Godeas A. M. Scervino J. M. (2015). The co-existence between DSE and AMF symbionts affects plant P pools through P mineralization and solubilization processes. *Fungal Ecol.* 17 10–17. 10.1016/j.funeco.2015.04.004

[B12] Deng J. Ding G. Gao G. Wu B. Zhang Y. Qin S. (2015). The sap flow dynamics and response of *Hedysarum scoparium* to environmental factors in semiarid northwestern China. *PLoS One* 10:e0131683. 10.1371/journal.pone.0131683 26136229 PMC4489904

[B13] Elavarthi S. Martin B. (2010). “Spectrophotometric assays for antioxidant enzymes in plants,” in *Plant Stress Tolerance: Methods and Protocols*, ed. Sunkar R. (Totowa, NJ: Humana Press), 273–280. 10.1007/978-1-60761-702-0_16 20387052

[B14] Fan B. Zhang A. Yang Y. Ma Q. Li X. Zhao C. (2016). Long-term effects of xerophytic shrub *Haloxylon ammodendron* plantations on soil properties and vegetation dynamics in Northwest China. *PLoS One* 11:e0168000. 10.1371/journal.pone.0168000 27992458 PMC5161353

[B15] Gong C. Wang J. Hu C. Wang J. Ning P. Bai J. (2015). Interactive response of photosynthetic characteristics in *Haloxylon ammodendron* and *Hedysarum scoparium* exposed to soil water and air vapor pressure deficits. *J. Environ. Sci.* 34 184–196. 10.1016/j.jes.2015.03.012 26257361

[B16] González-Teuber M. Urzúa A. Plaza P. Bascuñán-Godoy L. (2018). Effects of root endophytic fungi on response of *Chenopodium quinoa* to drought stress. *Plant Ecol.* 219 231–240. 10.1007/s11258-017-0791-1

[B17] González-Teuber M. Vilo C. Bascuñángodoy L. (2017). Molecular characterization of endophytic fungi associated with the roots of *Chenopodium quinoa* inhabiting the Atacama Desert. Chile. *Genomics Data* 11 109–112. 10.1016/j.gdata.2016.12.015 PMC5233788 28116242

[B18] Hu X. W. Wang Y. R. Wu Y. P. (2009). Effects of the pericarp on imbibition, seed germination, and seedling establishment in seeds of *Hedysarum scoparium* Fisch. et Mey. *Ecol. Res.* 24 559–564. 10.1007/s11284-008-0524-y

[B19] Jin H. Q. Liu H. B. Xie Y. Y. Zhang Y. G. Xu Q. Q. Mao L. J. (2018). Effect of the dark septate endophytic fungus *Acrocalymma vagum* on heavy metal content in tobacco leaves. *Symbiosis* 74 89–95. 10.1007/s13199-017-0485-4

[B20] Jumpponen A. Trappe J. M. (1998). Dark septate endophytes: a review of facultative biotrophic root-colonizing fungi. *New Phytol.* 140 295–310. 10.1046/j.1469-8137.1998.00265.x 33862835

[B21] Khan M. M. Islam E. Irem S. Akhtar K. Ashraf M. Y. Iqbal J. (2018). Pb-induced phytotoxicity in para grass (*Brachiaria mutica*) and castorbean (*Ricinus communis* L.) antioxidant and ultrastructural studies. *Chemosphere* 200 257–265. 10.1016/j.chemosphere.2018.02.101 29494906

[B22] Khastini R. O. Ohta H. Narisawa K. (2012). The role of a dark septate endophytic fungus, Veronaeopsis simplex Y34, in Fusarium disease suppression in Chinese cabbage. *J. Microbiol.* 50 618–624. 10.1007/s12275-012-2105-6 22923110

[B23] Kivlin S. N. Emery S. M. Rudgers J. A. (2013). Fungal symbionts alter plant responses to global change. *Am. J. Bot.* 100 1445–1457. 10.3732/ajb.1200558 23757444

[B24] Knapp D. G. Kovács G. M. Zajta E. Groenewald J. Z. Crous P. W. (2015). Dark septate endophytic pleosporalean genera from semiarid areas. *Persoonia* 35 87–100. 10.3767/003158515X687669 26823630 PMC4713113

[B25] Knapp D. G. Pintye A. Kovács G. M. (2012). The dark side is not fastidious–dark septate endophytic fungi of native and invasive plants of semiarid sandy areas. *PLoS One* 7:e32570. 10.1371/journal.pone.0032570 22393417 PMC3290574

[B26] Li B. He X. He C. Chen Y. Wang X. (2015). Spatial dynamics of dark septate endophytes and soil factors in the rhizosphere of *Ammopiptanthus mongolicus* in inner mongolia. China. *Symbiosis* 65 75–84. 10.1007/s13199-015-0322-6

[B27] Li L. Chen X. Shi L. Wang C. Fu B. Qiu T. (2017). A proteome translocation response to complex desert stress environments in perennial *Phragmites* sympatric ecotypes with contrasting water availability. *Front. Plant Sci.* 8:511. 10.3389/fpls.2017.00511 28450873 PMC5390029

[B28] Li T. Liu M. J. Zhang X. T. Zhang H. B. Sha T. Zhao Z. W. (2011). Improved tolerance of maize (*Zea mays* L.) to heavy metals by colonization of a dark septate endophyte (DSE) Exophiala pisciphila. *Sci. Total Environ.* 409 1069–1074. 10.1016/j.scitotenv.2010.12.012 21195456

[B29] Li X. He X. Hou L. Ren Y. Wang S. Su F. (2018). Dark septate endophytes isolated from a xerophyte plant promote the growth of *Ammopiptanthus mongolicus* under drought condition. *Sci. Rep.* 8:7896. 10.1038/s41598-018-26183-0 29785041 PMC5962579

[B30] Likar M. Regvar M. (2013). Isolates of dark septate endophytes reduce metal uptake and improve physiology of *Salix caprea* L. *Plant Soil* 370 593–604. 10.1007/s11104-013-1656-6

[B31] Lugo M. A. Molina M. G. Crespo E. M. (2009). Arbuscular mycorrhizas and dark septate endophytes in bromeliads from South American arid environment. *Symbiosis* 47 17–21. 10.1007/s11104-013-1656-6

[B32] Lugo M. A. Reinhart K. O. Menoyo E. Crespo E. M. Urcelay C. (2015). Plant functional traits and phylogenetic relatedness explain variation in associations with root fungal endophytes in an extreme arid environment. *Mycorrhiza* 25 85–95. 10.1007/s00572-014-0592-5 24997550

[B33] Mandyam K. Jumpponen A. (2005). Seeking the elusive function of the root-colonising dark septate endophytic fungi. *Stud. Mycol.* 53 173–189. 10.3114/sim.53.1.173

[B34] Newsham K. K. (2011). A meta-analysis of plant responses to dark septate root endophytes. *New Phytol.* 190 783–793. 10.1111/j.1469-8137.2010.03611.x 21244432

[B35] Perez-Naranjo J. C. (2009). *Dark Septate and Arbuscular Mycorrhizal Fungal Endophytes in Roots of Prairie Grasses.* Ph.D. Dissertation, Saskatoon: University of Saskatchewan.

[B36] Phillips J. M. Hayman D. S. (1970). Improved procedures for clearing roots and staining parasitic and vesicular-arbuscular mycorrhizal fungi for rapid assessment of infection. *Trans. Br. Mycol. Soc.* 55 158–163. 10.1016/S0007-1536(70)80110-3

[B37] Porras-Alfaro A. Herrera J. Sinsabaugh R. L. Odenbach K. J. Lowrey T. Natvig D. O. (2008). Novel root fungal consortium associated with a dominant desert grass. *Appl. Environ. Microb.* 74 2805–2813. 10.1128/aem.02769-07 18344349 PMC2394874

[B38] Ren A. Z. Li X. Han R. Yin L. J. Wei M. Y. Gao Y. B. (2011). Benefits of a symbiotic association with endophytic fungi are subject to water and nutrient availability in *Achnatherum sibiricum*. *Plant Soil* 346:363 10.1007/s11104-011-0824-9

[B39] Saleem M. Asghar H. N. Zahir Z. A. Shahid M. (2018). Impact of lead tolerant plant growth promoting rhizobacteria on growth, physiology, antioxidant activities, yield and lead content in sunflower in lead contaminated soil. *Chemosphere* 195 606–614. 10.1016/j.chemosphere.2017.12.117 29278850

[B40] Santos S. G. D. Silva P. R. A. D. Garcia A. C. Zilli J. É Berbara R. L. L. (2017). Dark septate endophyte decreases stress on rice plants. *Brazilian*. *J. Microbiol.* 48 333–341. 10.1016/j.bjm.2016.09.018 28089614 PMC5470451

[B41] Shi Z. Mickan B. Feng G. Chen Y. (2015). Arbuscular mycorrhizal fungi improved plant growth and nutrient acquisition of desert ephemeral *Plantago minuta* under variable soil water conditions. *J. Arid. Land.* 7 414–420. 10.1007/s40333-014-0046-0

[B42] Su Y. Z. Zhao W. Z. Su P. X. Zhang Z. H. Wang T. Ram R. (2007). Ecological effects of desertification control and desertified land reclamation in an oasis–desert ecotone in an arid region: a case study in hexi corridor, northwest China. *Ecol. Eng.* 29 117–124. 10.1016/j.ecoleng.2005.10.015

[B43] Su Z. Z. Mao L. J. Li N. Feng X. X. Yuan Z. L. Wang L. W. (2013). Evidence for biotrophic lifestyle and biocontrol potential of dark septate endophyte *Harpophora oryzae* to rice blast disease. *PLoS One* 8:e61332. 10.1371/journal.pone.0061332 23637814 PMC3630206

[B44] Surono Narisawa K. (2017). The dark septate endophytic fungus *Phialocephala fortinii* is a potential decomposer of soil organic compounds and a promoter of *Asparagus officinalis* growth. *Fungal Ecol.* 28 1–10. 10.1016/j.funeco.2017.04.001

[B45] Tang Z. An H. Deng L. Wang Y. Zhu G. Shangguan Z. (2016). Effect of desertification on productivity in a desert steppe. *Sci. Rep.* 6:27839. 10.1038/srep27839 27297202 PMC4906523

[B46] Vergara C. Araujo K. E. C. Urquiaga S. Santa-Catarina C. Schultz N. Araujo E. D. S. (2018). Dark septate endophytic fungi increase green manure-N-15 recovery efficiency, N contents, and micronutrients in rice grains. *Front. Plant Sci.* 9:613. 10.3389/fpls.2018.00613 29780402 PMC5946629

[B47] Wang J. L. Li T. Liu G. Y. Smith J. M. Zhao Z. W. (2016). Unraveling the role of dark septate endophyte (DSE) colonizing maize (*Zea mays*) under cadmium stress: physiological, cytological and genic aspects. *Sci. Rep.* 6:22028. 10.1038/srep22028 26911444 PMC4766571

[B48] Wang X. P. Zhang Y. F. Hu R. Pan Y. X. Berndtsson R. (2012). Canopy storage capacity of xerophytic shrubs in Northwestern China. *J. Hydrol.* 45 152–159. 10.1016/j.jhydrol.2012.06.003

[B49] Wilcox H. E. Wang C. J. K. (1987). Ectomycorrhizal and ectendomycorrhizal associations of Phialophorafinlandia with *Pinus resinosa*, *Picear ubens*, and *Betula alleghaniensis*. *Can. J. Res.* 17 976–990. 10.1139/x87-152 18685871

[B50] Wu L. Q. Lv Y. L. Meng Z. X. Chen J. Guo S. X. (2010). The promoting role of an isolate of dark-septate fungus on its host plant *Saussurea involucrata* Kar. et Kir. *Mycorrhiza* 20 127–135. 10.1007/s00572-009-0268-8 19707800

[B51] Xie L. (2017). *Species Diversity and Salt Tolerance of DSE in The Roots of Hedysarum Scoparium Fisch. et Mey. in Northwest China.* Ph.D. Dissertation, Baoding: Hebei University.

[B52] Xie L. He X. Wang K. Hou L. Sun Q. (2017). Spatial dynamics of dark septate endophytes in the roots and rhizospheres of *Hedysarum scoparium* in northwest China and the influence of edaphic variables. *Fungal Ecol.* 26 135–143. 10.1016/j.funeco.2017.01.007

[B53] Xie W. Hao Z. Zhou X. Jiang X. Xu L. Wu S. (2018). Arbuscular mycorrhiza facilitates the accumulation of glycyrrhizin and liquiritin in *Glycyrrhiza uralensis* under drought stress. *Mycorrhiza* 28 285–300. 10.1007/s00572-018-0827-y 29455337

[B54] Zhang Q. M. Gong M. G. Yuan J. F. Hou Y. Zhang H. M. Wang Y. (2017). Dark septate endophyte improves drought tolerance in *Sorghum*. *Int. J. Agric. Biol.* 19 53–60. 10.17957/ijab/15.0241

[B55] Zhu Z. D. Chen G. T. (1994). *Land Sandy Desertification in China.* Beijing: Science Press.

