# Supplementary material for: Effects of Dark Septate Endophytes on the Performance of Hedysarum scoparium Under Water Deficit Stress
Source: Front Plant Sci. 2019 Jul 11;10:903. doi: 10.3389/fpls.2019.00903 (PMC6637391; doi:10.3389/fpls.2019.00903)

Supplementary Material

Effects of Dark Septate Endophyte on the Performance of *Hedysarum scoparium* Under Water Deficit Stress

Xia Li, Xue-Li He^*^, Yong Zhou, Yi-Ting Hou, Yi-Ling Zuo

*** Correspondence:** Xue-Li He: xlh3615@126.com

## Supplementary Figures

**Supplementary Figure 1.** Effects of dark septate endophyte (DSE) inoculation on the biomass production of *Hedysarum scoparium*. E– indicates non-inoculated plants. CO and PA indicate plants inoculated with *Cladosporium oxysporum* and *Paraphoma* sp., respectively. These two DSE strains were isolated from the healthy roots of *H*. *scoparium* in the natural habitat. The plants of *H*. *scoparium* were growing under well-watered condition (70% field capacity) for three months. The error bars represent the standard error (SE). Different letters above the error bars indicate significant difference at *P* < 0.05 by Duncan’s multiple-range tests.


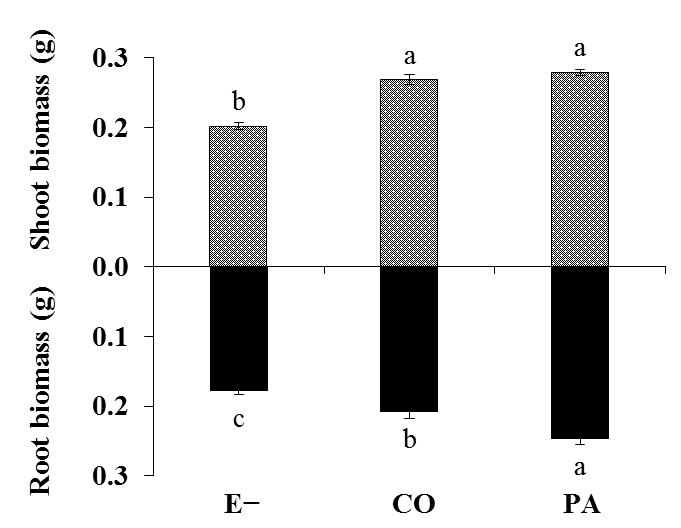


**Supplementary Figure 2.** Colonization of dark septate endophyte (DSE) strains in the roots of *Hedysarum scoparium* five months after inoculation. A, B, C, and D indicate roots inoculated with *Phialophora* sp., *Knufia* sp., *Leptosphaeria* sp., and *Embellisia chlamydospora*, respectively.


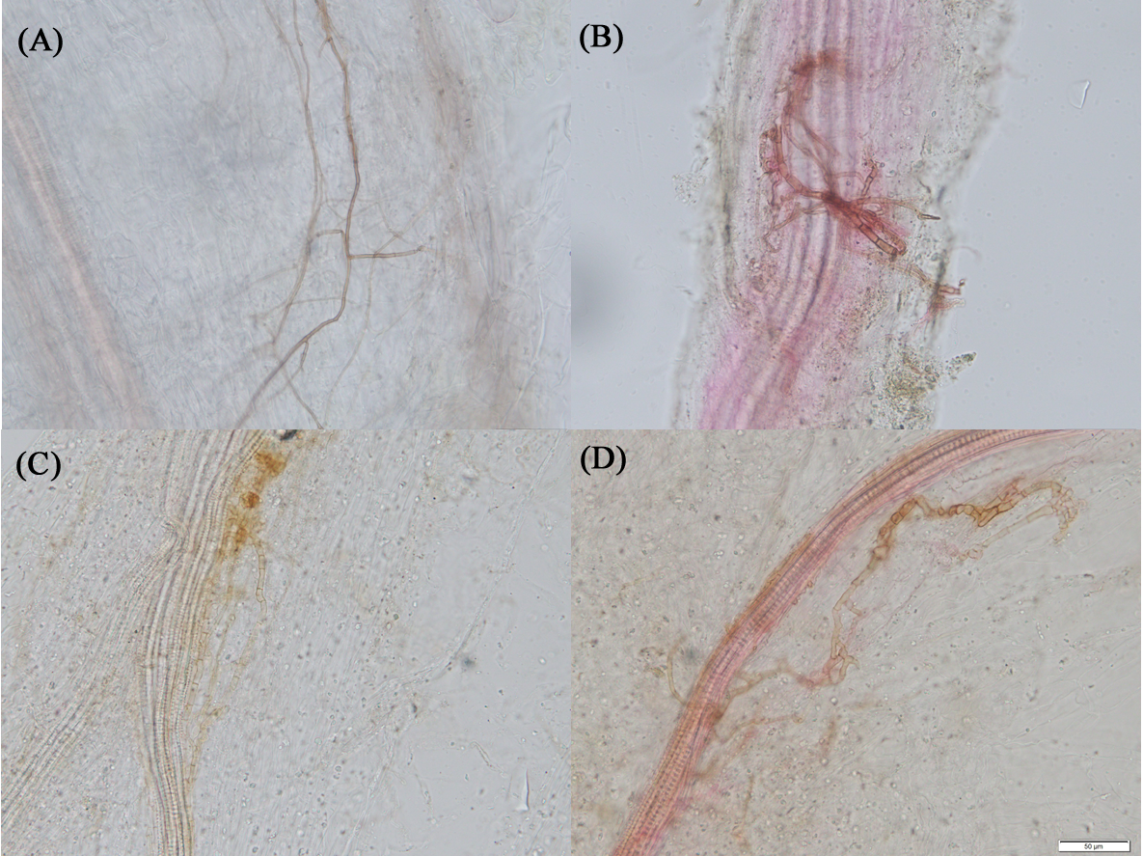

Supplement: Supplementary file 1 [file Data_Sheet_1.docx]
